# Tribochemical Interactions between Carbon Nanotubes and ZDDP Antiwear Additive during Tribofilm Formation on Uncoated and DLC-Coated Steel

**DOI:** 10.3390/ma13102409

**Published:** 2020-05-23

**Authors:** Wojciech Dzięgielewski, Joanna Kowalczyk, Andrzej Kulczycki, Monika Madej, Dariusz Ozimina

**Affiliations:** 1 Department Propellants & Lubricants Devision, Air Force Institute of Technology, 01-494 Warszawa, Poland; wojciech.dziegielewski@itwl.pl (W.D.); andrzej.kulczycki@itwl.pl (A.K.); 2 Department of Mechatronics and Mechanical Engineering, Kielce University of Technology, 25-314 Kielce, Poland; mmadej@tu.kielce.pl (M.M.); ozimina@tu.kielce.pl (D.O.)

**Keywords:** tribological process, lubricant, surface layers of solid elements, zinc dithiophosphate, carbon nanotubes

## Abstract

The data from the authors’ earlier investigations show that molecules of zinc dithiophosphate (ZDDP) added to a lubricant can absorb energy emitted by a solid surface, which is where triboreactions occur. If the lubricant contains structures able to conduct energy, the ZDDP reactions can occur even at a relatively large distance from the solid surface, which should increase the effectiveness of ZDDP as an antiwear additive. The purpose of this paper was to verify the thesis that the tribocatalytic effect depends on the ability of the solid surface to emit electrons/energy and the ability of ordered molecular structures, such as carbon nanotubes (CNTs), to conduct energy and, most likely, to enhance the energy transfer. The tribological tests were performed using a TRB^3^ tribotester for 100Cr6 steel balls and uncoated or a-C:H coated HS6-5-2C steel discs. Polyalphaolefin 8 (PAO8) and PAO8 mixed with ZDDP and CNTs were used as lubricants. The results of the tribological tests suggested that: (a) the effect of the interactions between ZDDP and CNTs was clearly visible; (b) the structure and properties of the solid surface layer had a significant influence on the antiwear action of the ZDDP additive.

## 1. Introduction

Lubricants containing zinc dithiophosphates (ZDDPs) as antiwear additives have been used for decades. Various mechanisms of lubricating film formation have been developed and described, but most of them assume that molecules of a ZDDP additive undergo adsorption on the solid surface, and the chemical reactions lead to the formation of deposits on the solid surface, as well as the formation of reaction products inside the surface layer. All the products of ZDDP reactions are considered to be responsible for lower values of the friction coefficient and better protection of solid elements against wear. Because of this mechanism, ZDDP reactions take place on the surfaces of the solid elements in contact. This assumption is consistent with the definition of tribochemistry because tribochemistry is “the chemical reactions that occur between the lubricant/environment and the surfaces under boundary lubrication conditions” [1]. We do not agree with this definition, claiming that, if the lubricant is treated as the environment, the lubricating film does not form in the tribological system; thus, none of the processes occurring inside the thin lubricating film should be taken into account. It is well known, however, that the physical and chemical properties of the lubricant play a significant role under boundary or mixed lubrication conditions. The lubricant cannot be treated as a dissolvent of lubricating additives, such as ZDDP.

The results of the authors’ earlier research [2,3] clearly indicate how important the properties of lubricants are in the formation of a lubricating film. The tribological tests, carried out using a high-frequency reciprocating rig (HFRR) [4], reveal that the presence of an antistatic additive (ASA) affects the effectiveness of ZDDP, decreasing the coefficient of friction and wear and, what is more, increasing the thickness of the lubricating film. Commercial ASA is an additive used in aircraft jet fuels to increase their electrical conductivity.

The ASA is composed of C/H/O/S polymer, polyamine, and R-SO_3_H as a stabilizer. The chemical structure of the ASA indicates that its components can form ordered molecular structures so that, even at a very low concentration (6 ppm), it can substantially increase the electrical conductivity of the lubricant, and as a result, enhance the antiwear action of ZDDP to form a lubricating film [4].

The results of the authors’ previous research, described in [2,3], were used to formulate a concept of the mechanism of lubricating film formation. The postulated mechanism assumes interactions between the ZDDP molecules, the molecules of the other lubricant components, capable of energy transfer (e.g., the ASA additive), and the material of the solid elements in contact. It should be emphasized that all the elements of the tribological system are treated as equally important.

This mechanism is consistent with the literature on the role of carbon molecular clusters, such as polyaromatic hydrocarbon (PAH) clusters, in the transport of electrons [4,5,6,7,8,9] and their emission due to friction [10,11].

The tribocatalytic mechanism proposed in [4,12] assumes that the surface of a solid element in contact with another solid element is the donor of electrons and/or photons. Since molecules of a lubricating additive are acceptors of electrons/energy, structures able to conduct energy from the solid surface to the additive molecules are required. From the earlier investigations, it is apparent that ZDDP molecules can absorb energy when they are on or near the solid surface. Thus, the ZDDP reactions take place on the solid surface. In the case of lubricant-containing structures able to conduct energy, the ZDDP reactions can occur even at a relatively large distance from the solid surface.

The proposed mechanism of tribocatalysis indicates that the tribocatalytic effect depends on the ability of the solid surface to emit electrons/energy and the ability of the ordered molecular structures to conduct energy and probably enhance its transfer. Carbon nanotubes (CNTs), which are now being extensively studied [13], have incredible electrical, mechanical [13,14], thermal, and chemical properties and, as such, can be used in many industrial sectors, including nanoelectronics, biotechnology, and thermal management [13]. Over the last few years, there have been many papers on the tribological properties of CNTs, fullerenes, and graphene. Some of them, e.g., [15], focus on superlubricity. Sharmin et al. [16] compared conventional cutting fluid with oil mixed in water with CNT based nano-fluid. The use of CNT-based nano-fluid contributed to lower surface roughness and lower cutting temperature (up to 34% and 29%, respectively) compared to the conventional cutting fluid. It was concluded that the sample with 0.3 vol. % CNTs had a great potential to be used as an efficient coolant and lubricant in machining because of its higher stability, greater thermal conductivity, and lower viscosity.

Salah et al. [17] tested a lubricant containing CNTs dispersed in sunflower oil in a concentration of 0.005–0.5 wt. % The tests, performed using a ball-on-disk tribometer, revealed that the friction coefficient was around 58% lower than that for the lubricant without any additives. This was true even at a very low concentration of CNTs (about 0.1 wt. %). The friction coefficient was assumed to decrease with increasing load due to the formation of protective graphitic carbon layers on the surfaces in contact.

In the literature on tribological systems, it is difficult to find any information, including experimental data, on the influence of CNTs on the interaction between the surfaces of solid elements and lubricating additives. The purpose of this paper was to examine the impact of the surface layers of the solid elements in contact on the effectiveness of carbon clusters (CNTs) in enhancing the action of ZDDP as a lubricating additive.

## 2. Materials and Methods

### 2.1. Materials

The tribological tests were carried out to determine the role of ordered carbon structures in the tribocatalytic effect. The base oil used to prepare the different lubricants was polyalphaolefin 8 (PAO8), a commercially available synthetic oil with a hydrocarbon (isoparaffin) structure, produced by catalytic oligomerization of linear α-olefins containing 8–12 carbon atoms in the chain. The properties of PAO8 are provided in Table 1.

Table 2 shows the properties of the ZDDP antiwear additive added to PAO8 at a concentration, used in commercial lubricants of 1.5% (m/m), to be tested tribologically.

The tribological tests were performed for the materials listed in Table 3.

The carbon nanotubes used in the experiments were a commercial product made by CARBON4nano (Institute of Carbon Technologies, Toruń, Poland). They are industrial grade single-walled, unmodified carbon nanotubes with an average diameter of 1.6 nm and a length of 5 µm. The carbon purity of the product exceeds 85% [18]; the impurities have organic substituents containing O, Al, and Fe, which are bonded with the carbon atoms of the carbon nanotubes. The CNTs were dispersed in PAO8 and in PAO8 mixed with ZDDP, without dispersants, using high-speed homogenization. The presence of dispersants generally ensures a durable dispersion of CNTs in PAO oil [19]. The authors’ experience has shown that small concentrations of CNTs, e.g., 0.005% (m/m), guarantee a durable suspension with no agglomerates. This concentration is sufficient to effectively help ZDDP improve the antiwear behavior of a lubricant. Basing on the results of tribological tests described in [17], the 0.005% (m/m) concentration of CNTs was indicated as optimal. No dispersants were used in the experiments to prevent disturbance to the interaction between the additives. The dispersions of CNTs prepared using a high-speed homogenizer were durable throughout the tribological tests. 

The a-C:H coating was deposited on HS6-5-2C high-speed steel, characterized by very good ductility, high impact strength, and high wear resistance. The chemical composition of HS6-5-2C is given in Table 4.

The material was designed to operate well at elevated temperatures; it could be heat treated at high temperatures: quenching at 1190–1230 °C and tempering at 550–650 °C. After heat treatment at 500–550 °C, its hardness reached 65 HRC.

### 2.2. Equipment and Methodology

The tribological tests were performed by means of a tribotester (TRB^3^, Anton Paar TriTec, Neuchatel, Switzerland) operating in a ball-on-disc configuration. The experiments were carried out under the following conditions:

- load P = 10 N,
- sliding speed v = 0.1 m/s,
- sliding distance s = 1000 m,
- humidity 25 ± 5%,
- ambient temperature T_0_ = 25 ± 4 °C,
- elements in contact: 100Cr6 steel balls and HS6-5-2C steel discs, with the latter uncoated or coated with a-C:H.

The 100Cr6 steel balls were 6 mm in diameter. The initial Hertzian contact pressure in the steel ball and steel disc system was 1.93 GPa, as recorded by the TRB^3^ tribological tester software.

Each lubricant was tested twice, i.e., for an uncoated disc and for a disc coated with a-C:H. Diamond-like carbon (DLC) coatings have excellent tribological properties, good corrosion resistance, and high hardness. This is the reason why a-C:H coatings are used in so many industrial applications [20,21]. In addition to their exceptional mechanical and tribological properties, diamond-like carbon coatings are characterized by low and negative electron affinity. DLC coatings have properties similar to those of diamond [22].

The diamond-like carbon coatings were studied with a Nicolet Almega XR Raman spectrometer (Thermo Electron Scientific Instruments LLC, Madison, WI, USA) using a 532 nm Nd:YAG laser as an excitation source. A power of 2.5 mW was applied in the measurements. 

**Figure 1:**
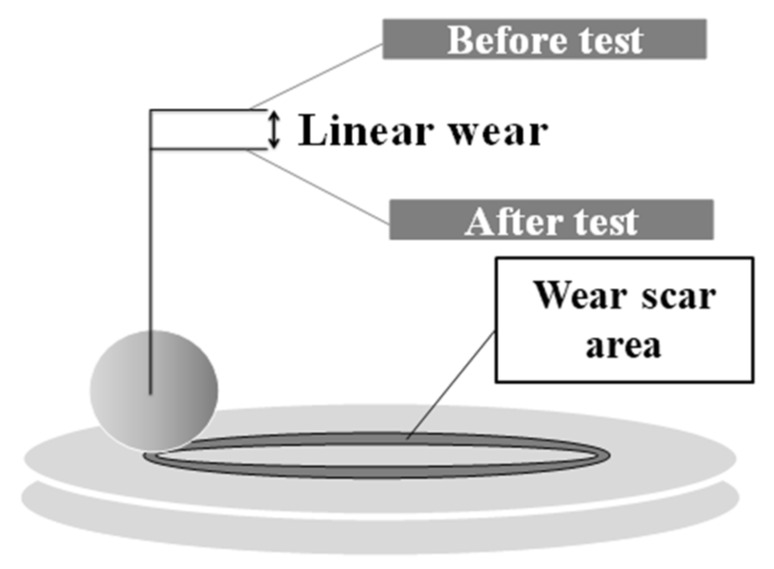
Linear wear and wear scar area registered to analyze the role of lubricants in the formation of an antiwear film.

The tests involved measuring the following tribological properties of the lubricants: the linear wear, the coefficient of friction, and the wear scar area. The measurement of the linear wear and the wear scar area is illustrated in Figure 1.

The lubricating film forming in the ball-on-disc tribological system was 0.06 µm in thickness. The wear of the ball and the disc indicated that the ball-disc system operated under boundary lubrication conditions. The wear tracks on the discs and the wear scars on the balls were examined using a confocal interferometric microscope (Leica Microsystems, DCM8, Wetzlar, Germany). The measurement and analysis of the surface textures were performed before and after each friction test. A scanning electron microscope equipped with an EDS microanalyzer (Phenol XL, Thermo Fisher Scientific, Eindhoven, The Netherlands) was used to determine the concentrations of elements for each specimen before and after the tribological test.

## 3. Results

### 3.1. a-C:H Coated Disc

**Figure 2:**
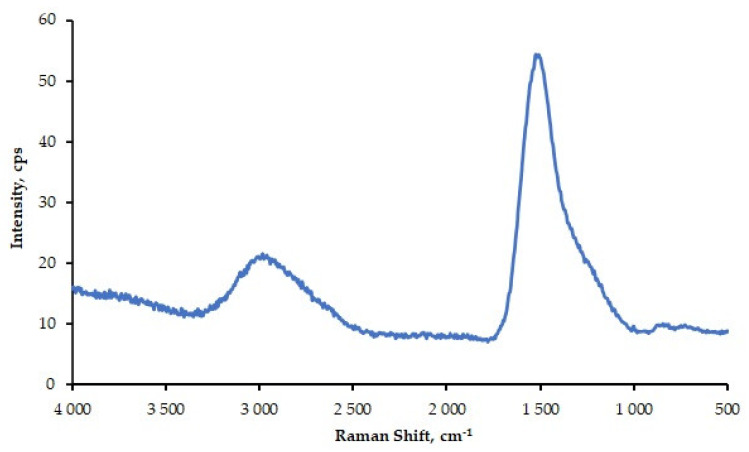
The Raman spectra of the diamond-like coating (DLC) coating.

The Raman spectra, recorded in the range of 100–4000 cm^−1^, with an actual resolution of approx. 6 cm^−1^, are shown in Figure 2.

**Figure 3:**
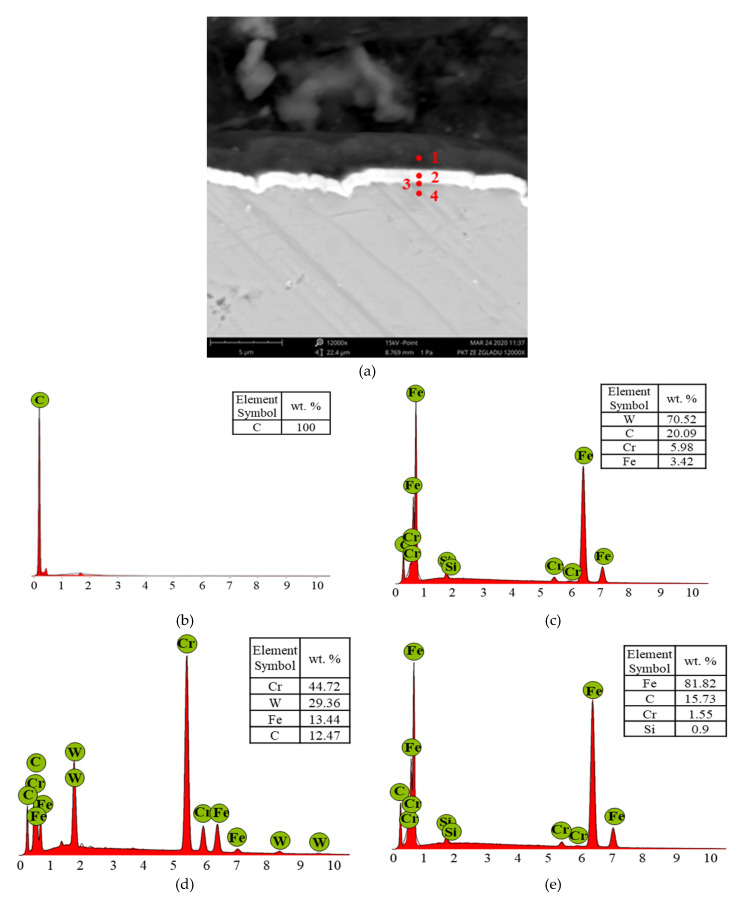
(**a**) A cross-sectional view of the disc structure with EDS patterns at four different points: (**b**)—the coating, (**c**) and (**d**)—the intermediate layer, (**e**)—the substrate.

The structure of the a-C:H coating is shown in Figure 3.

### 3.2. Tribological Data

**Figure 4:**
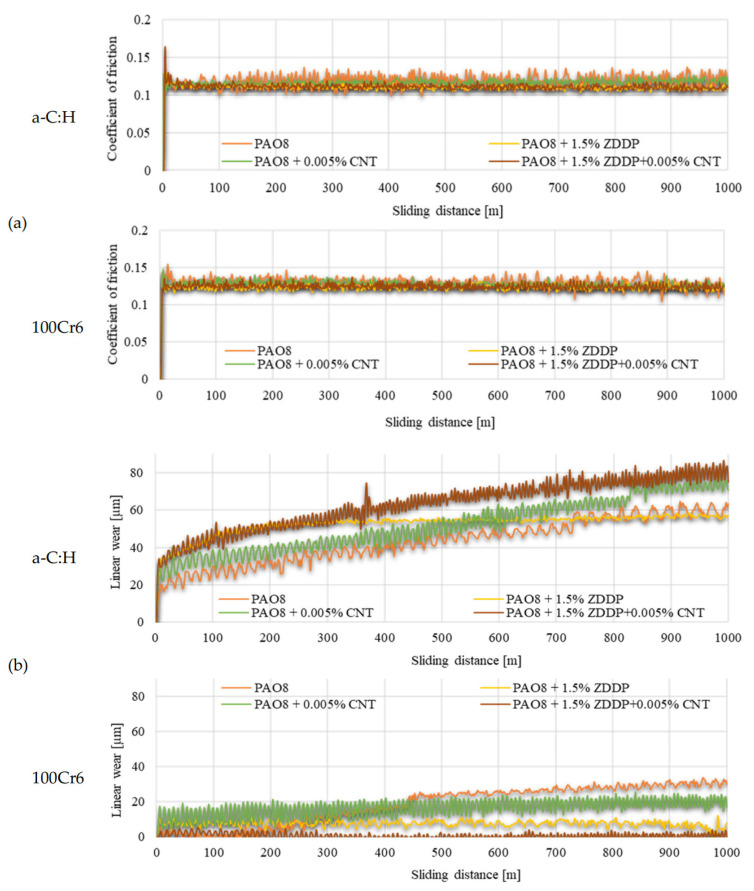
(**a**) Coefficient of friction vs. sliding distance, and (**b**) linear wear vs. sliding distance.

**Figure 5:**
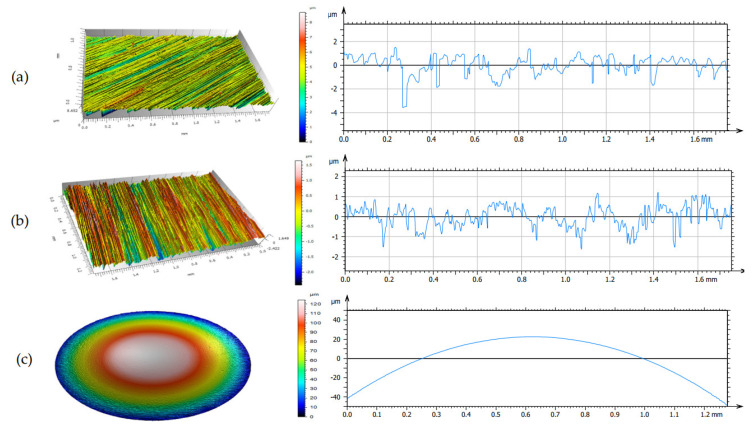
Surface textures before the tribological tests: isometric views and primary profiles of: (**a**) the a-C: H coated HS6-5-2C steel disc, (**b**) uncoated HS6-5-2C steel disc, (**c**) 100Cr6 steel ball.

**Figure 6:**
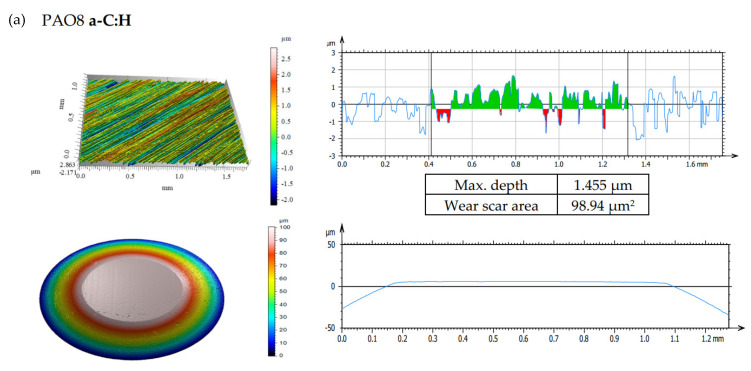

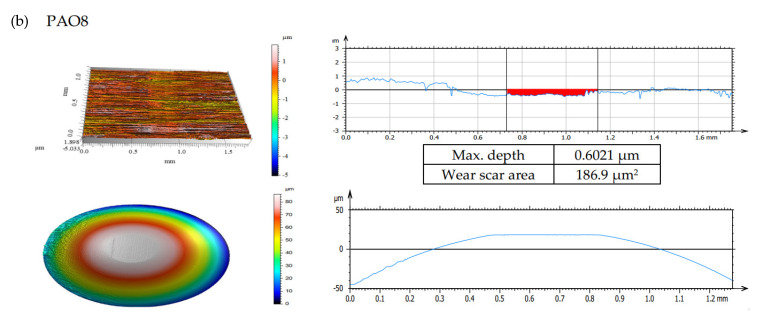
Isometric views and primary profiles of the discs and balls after the tribological tests of PAO8 on TRB^3^ tribotester equipped with (**a**) disc coated by a-C:H and (**b**) uncoated disc.

**Figure 7:**
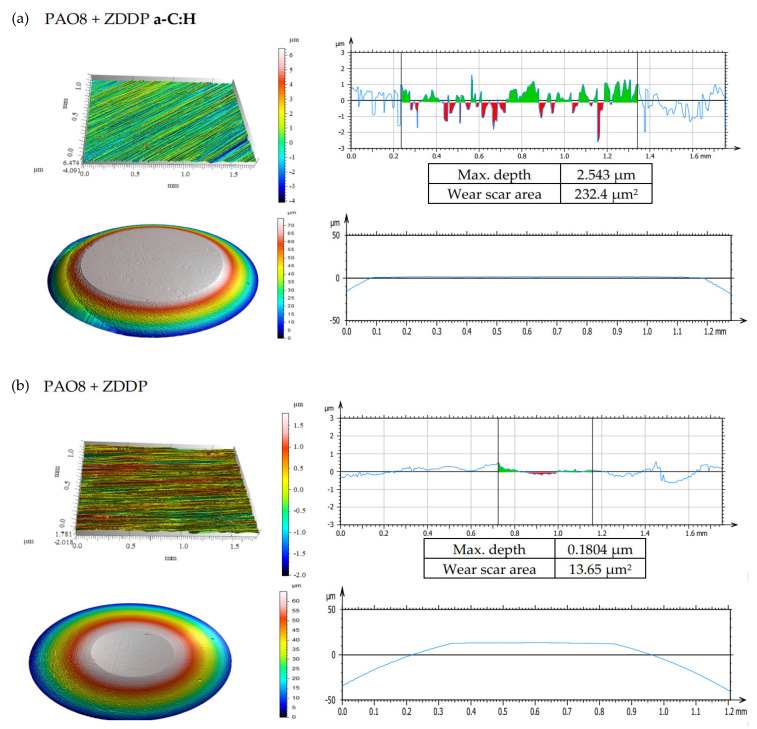
Isometric views and primary profiles of the discs and balls after the tribological tests of PAO8 + ZDDP on TRB^3^ tribotester equipped with (**a**) disc coated by a-C:H and (**b**) uncoated disc. ZDDP, zinc dithiophosphate.

**Figure 8:**
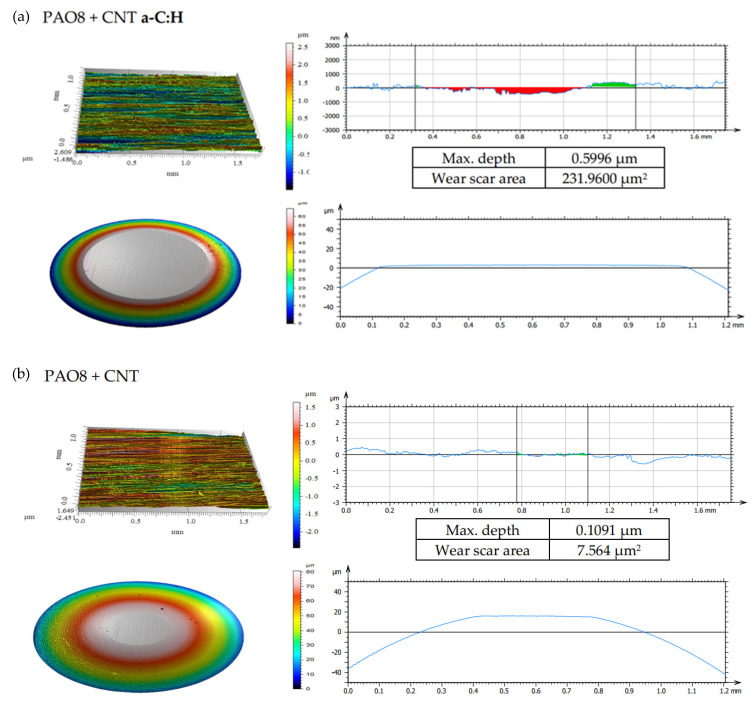
Isometric views and primary profiles of the discs and balls after the tribological tests of PAO8 + CNT on TRB^3^ tribotester equipped with (**a**) disc coated by a-C:H and (**b**) uncoated disc. CNT, carbon nanotube.

**Figure 9:**
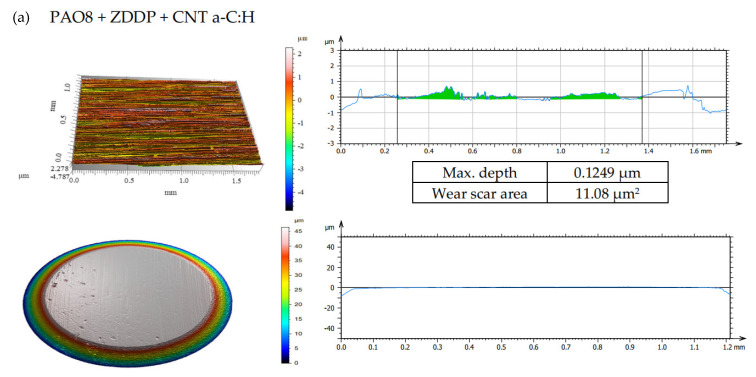

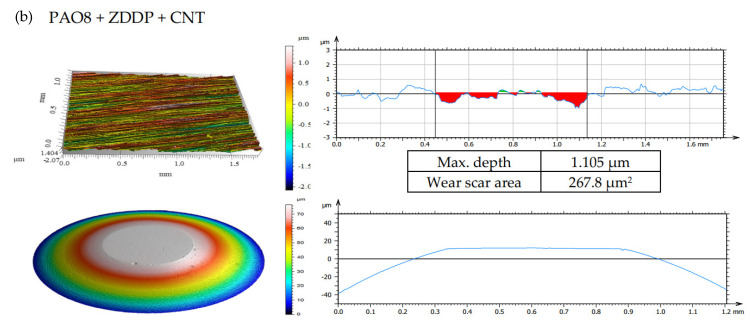
Isometric views and primary profiles of the discs and balls after the tribological tests of PAO8 + ZDDP + CNT on TRB^3^ tribotester equipped with (**a**) disc coated by a-C:H and (**b**) uncoated.

**Figure 10:**
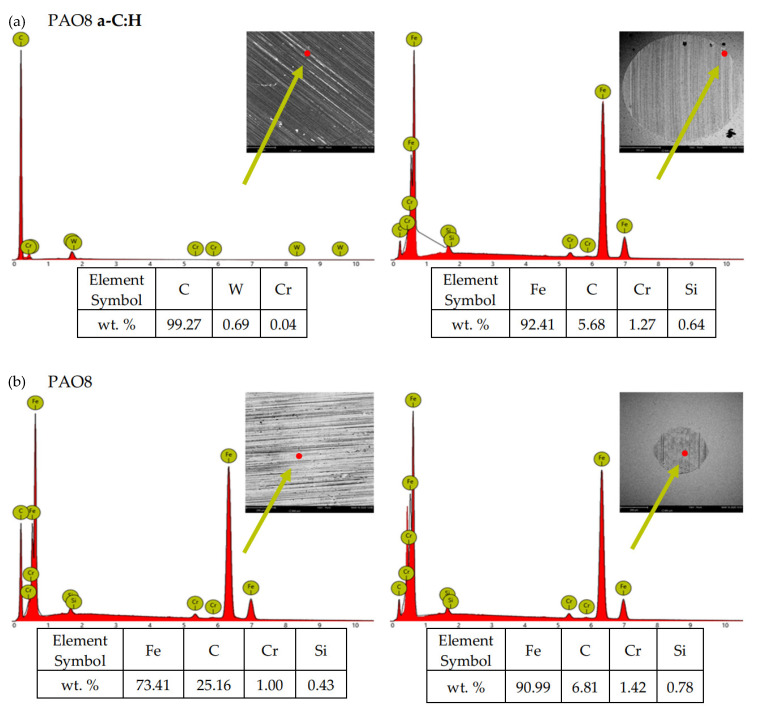
EDS patterns for different points of the wear track resulting from the sliding contact of the (**a**) coated and (**b**) uncoated steel discs with the steel balls—lubricant: PAO8.

**Figure 11:**
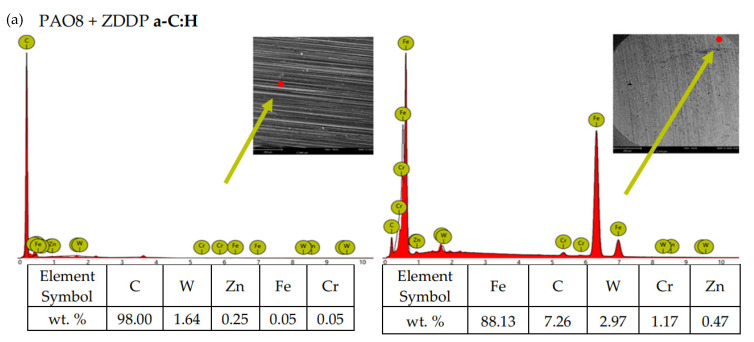

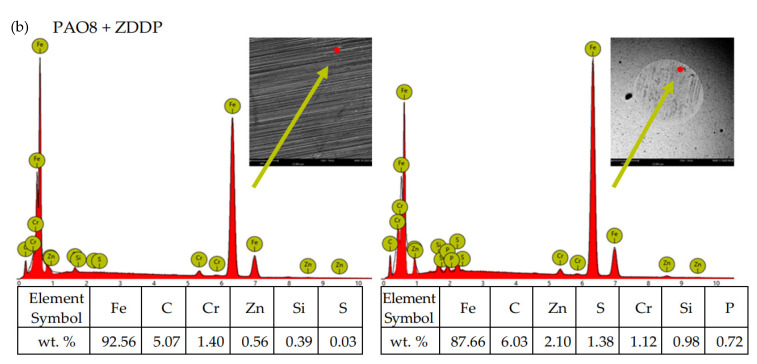
EDS patterns for different points of the wear track resulting from the sliding contact of the (**a**) coated and (**b**) uncoated steel discs with the steel balls—lubricant: PAO8 + ZDDP.

**Figure 12:**
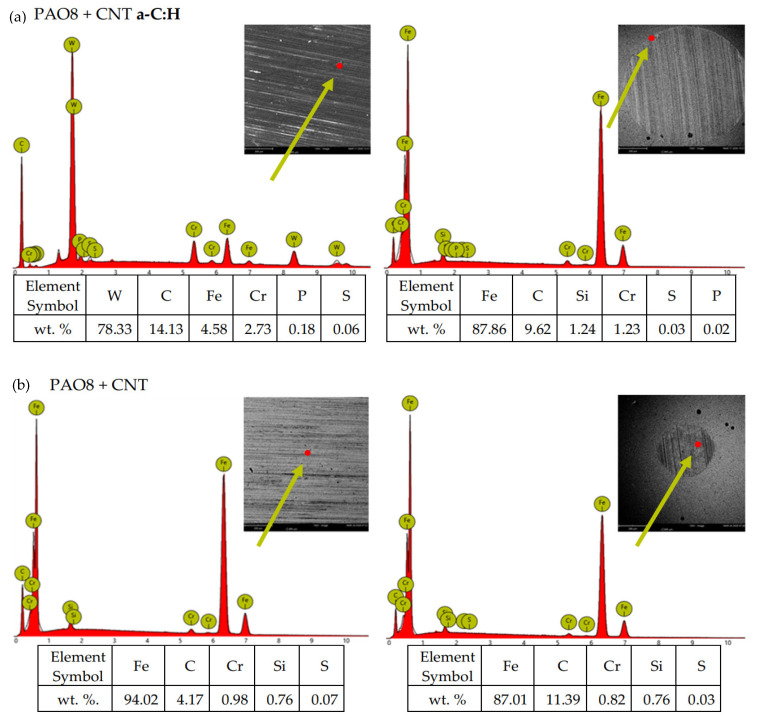
EDS patterns for different points of the wear track resulting from the sliding contact of the (**a**) coated and (**b**) uncoated steel discs with the steel balls—lubricant: PAO8 + CNT.

According to adopted methodology, each lubricant has been tested three times using TRB^3^ tribotester equipped with uncoated disc and three times using TRB^3^ tribotester equipped with a disc coated by a-C:H. It was found that the values of the results—coefficient of friction and linear wear—obtained for given lubricant and given kind of disc (uncoated or coated) were very similar. In this situation, one was chosen from the results obtained for each lubricant and each kind of disc. Figure 4 shows the coefficient of friction and linear wear as the results of the chosen tests. Balls and discs after these tests were analyzed. In Figure 5, Figure 6, Figure 7 and Figure 8, the isometric views and primary profiles of discs and balls are shown. Figure 9, Figure 10, Figure 11 and Figure 12 show the results of the EDS analysis of these balls and discs.

Figure 4 illustrates the relationships between the coefficient of friction and the sliding distance and between the linear wear and the sliding distance for the a-C:H coated disc and the steel ball.

In Figure 4a, there are shown the results of the friction coefficients measurement:

- top diagram for discs coated by a-C:H
- bottom diagram for uncoated steel discs.

In Figure 4b, there are shown the results of the linear wear measurement:

- top diagram for discs coated by a-C:H
- bottom diagram for uncoated steel discs.

Each diagram collects the results of the measurements obtained for all tested lubricants.

Figure 5 shows the isometric views and primary profiles obtained for the a-C:H coated and uncoated steel discs and steel balls before the tribological tests. From the isometric view and the primary profile of the a-C:H-coated HS6-5-2C steel, it was evident that the coated surface with peaks of approx. 1 µm and valleys of about 1.5 µm was smoother than the uncoated surface.

Figure 6, Figure 7, Figure 8 and Figure 9 show the isometric views and primary profiles obtained for the a-C:H coated and uncoated steel discs and steel balls after the tribological tests.

Table 5 provides the surface texture parameters recorded for the discs and balls before and after the tribological tests. The results obtained for the uncoated steel discs are given in Table 5a, while the data concerning the a-C:H coated discs are shown in Table 5b.

**Figure 13:**
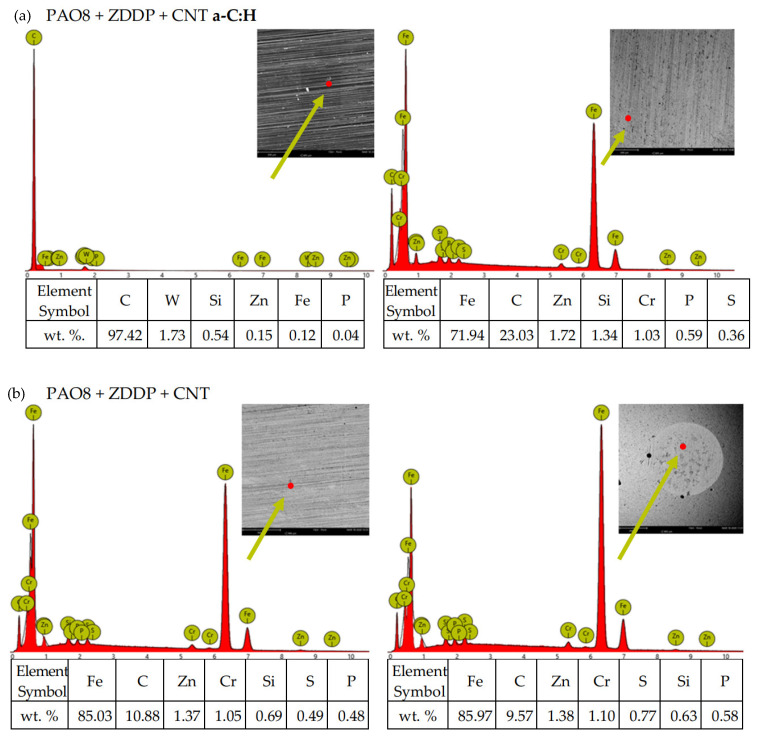
EDS patterns for different points of the wear track resulting from the sliding contact of the (**a**) coated and (**b**) uncoated steel discs with the steel balls—lubricant: PAO8 + ZDDP + CNT.

Figure 10, Figure 11, Figure 12 and Figure 13 show the results of the elemental analysis for different points on the wear track resulting from the sliding contact of the coated or uncoated HS6-5-2C steel discs with the 100Cr6 steel balls. The measurements involved determining the concentrations of C, W, and Cr as the elements of the a-C:H coating, Fe, C, Cr, and Si as the elements of the steel, and Zn, P, and S as the elements of the ZDDP additive.

## 4. Discussion

The analysis of the tribological data revealed that the structure and properties of the disc surface layer had considerable influence on the wear of both elements in contact. The character of wear (linear wear and wear scars) suggested that wear was dependent on the tribochemical activity of the lubricant components, i.e., lubricant additives.

The Raman spectra (Figure 2) were then fitted using a Gaussian profile to determine the positions and intensities (I) of the D and G peaks. The positions of the G and D peaks for the a-C:H coatings were 1570 cm^−1^ and 1364 cm^−1^, respectively. The ID/IG ratio was 0.87. These values were compared with the data provided by Ferrari and Robertson in [23]. The calculation results revealed that the analyzed DLC coatings had a high content of sp3 type bonds (approximately 30%). The position of the G peaks above 1550 cm^−1^ for the visible excitation Raman spectra also indicated a high content of sp3 type bonds.

The DLC coating consisted of carbon (Figure 3b). The upper part of the intermediate layer was tungsten, while the lower was chrome (Figure 3c,d, respectively). The surface and the intermediate layer seemed to improve the tribological properties of the disc material by reducing friction and wear and increasing the adhesion of the coating to the substrate material.

Figure 4a compares the values of the coefficient of friction obtained for the different tribological systems. The lowest value (approx. 0.11) was reported for the a-C:H-coated disc when the PAO8 + ZDDP + CNT was used. The highest value (approx. 0.12) was observed for the steel disc when the PAO8 + ZDDP + CNT was applied.

Figure 4b compares the values of the linear wear obtained for the different tribological systems. The lowest value (approx. 2 µm) was reported for the steel disc when the PAO8 + ZDDP + CNT was used. The highest value (approx. 80 µm) was observed for the a-C:H-coated disc when the same lubricant (PAO8 + ZDDP + CNT) was present.

The results shown in Figure 8b indicated that the presence of CNTs in the lubricant had a substantial influence on linear wear. It was found that when the discs were coated with a-C:H, ZDDP (the antiwear additive) promoted wear; in the presence of both ZDDP and CNTs, the wear was even higher. In the case of uncoated discs, however, the presence of ZDDP in the lubricant resulted in lower wear, and the addition of CNTs enhanced the antiwear action of ZDDP.

After the tribological tests in the presence of PAO8 + 0.005 CNT (Figure 8), the shallowest wear track of about 0.1 µm and a small wear scar area of about 7.56 µm^2^ were observed. The wear tracks on the discs coated with DLC were almost invisible to the naked eye; the wear tracks on the steel balls in contact with the coated discs were deeper than those on the balls in contact with the uncoated discs.

**Figure 14:**
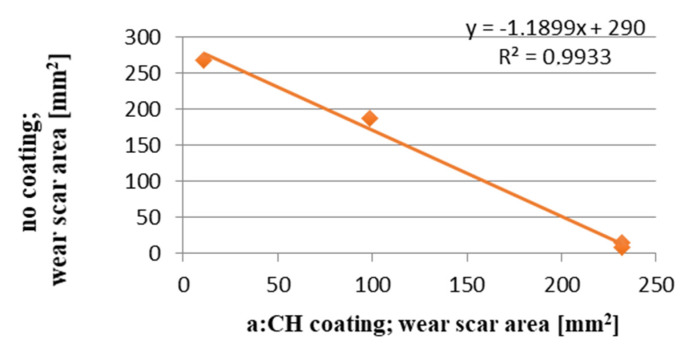
Wear scar area on the coated discs vs. wear scar area on the uncoated ones.

An inversely proportional relationship was found between the wear scar area on the a-C:H coated discs and that on the uncoated discs. The results illustrated in Figure 14 suggested that the coating “reversed” the mechanism of the antiwear action of the lubricant.

The analysis of the surface texture parameters obtained for the uncoated steel discs (Table 5a) revealed that the lowest values of Sa (arithmetical mean heigh), Sq (root mean square height), and Sp (maximum peak high) were reported for the PAO + ZDDP + CNT mixture; after the tribological test, the disc surface was smoothed.

When the surface texture parameters of a-C: H coated discs were studied (Table 5b), the lowest value of Sa was observed for the PAO8 + ZDDP lubricant. The PAO8 + CNT mixture, on the other hand, ensured the lowest values of Sq (root mean square height), Sv (maximum pit height), Sz (maxium height), Ssk (skewness), and Sku (kurtosis), which indicated the smoothing of the disc surface. Finally, the lowest value of the parameter Sp was obtained for PAO8 + ZDDP + CNT.

The parameter Ssk had a negative value in all the cases considered, which suggested a plateau-like surface with gently sloped and rounded peaks [24].

**Figure 15:**
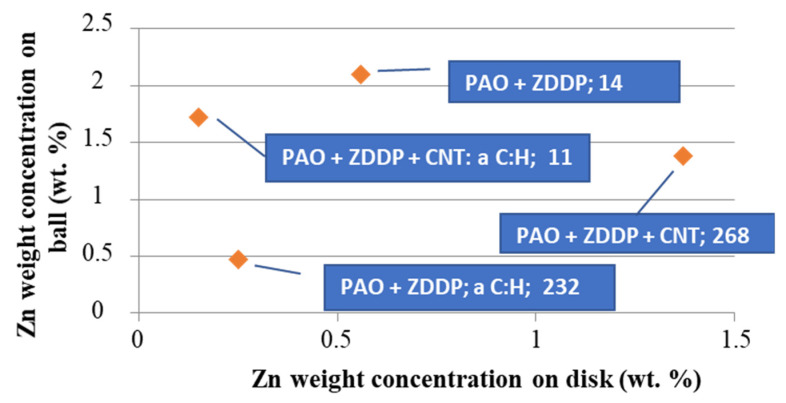
Relationship between the concentration of Zn on the ball and that on the disc after the tribological tests; the numbers provided correspond to the wear scar areas (μm^2^).

The results provided in Figure 6, Figure 7, Figure 8 and Figure 9 indicated differences in the wear between the discs and the balls, according to the lubricant composition. Different lubrication conditions might mean a different direction and/or different intensity of energy transfer in the ZDDP triboreactions. Figure 15 shows that:

- a high concentration of Zn on the ball surface gave a small wear scar area,
- even a high concentration of Zn on the disc surface did not protect it against wear.

**Figure 16:**
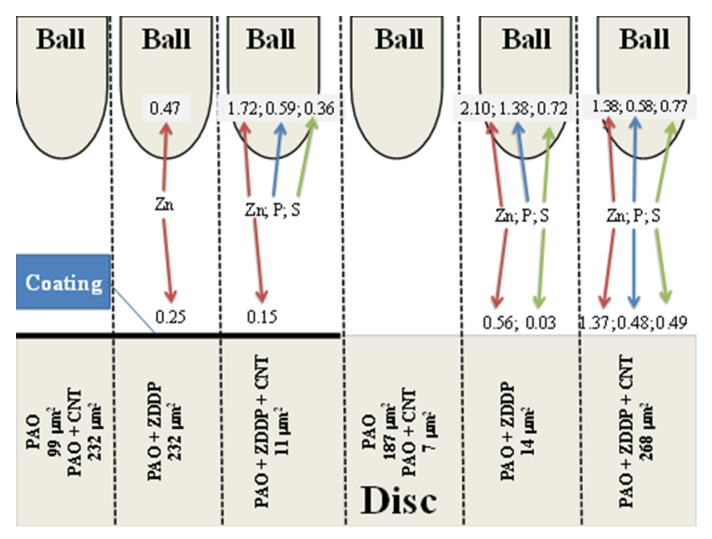
Influence of the coating on the discs and CNTs in the lubricant on the distribution of Zn-, P-, and S-containing products of the ZDDP triboreaction.

Figure 16 suggests that the wear scar area did not depend on the concentrations of products of the ZDDP triboreactions but on the relationship between the concentrations of these products on the ball surface and those on the disc surface. When the concentrations of the ZDDP triboreaction products on the ball surface were much higher, there was a decrease in the wear scar area. This could be due to energy transfer. CNTs, able to conduct energy but unable to form a protective layer, had the same effect as ZDDP. In the case of a-C:H coated discs, the addition of either CNTs or ZDDP led to a significant increase in the wear scar area, as compared with the effect of PAO8. The addition of both CNTs and ZDDP caused a rapid decrease in the wear scar area. An inverse situation took place when the discs were not coated.

From the analysis of the linear wear (Figure 4b), it was clear that: (a) the linear wear was generally higher on discs coated with an a-C:H than on uncoated ones; (b) in the case of a-C:H coated discs, the addition of CNTs or both CNTs and ZDDP to the lubricant caused a significant increase in the linear wear; (c) in the case of uncoated discs, the addition of CNTs or ZDDP, or CNTs and ZDDP together caused a significant decrease in the linear wear.

A similar study was carried out by Puhan and Reddyhoff [25]. They tested a lubricant containing a ZDDP additive using a pin-on-disc apparatus. The discs were coated with aluminum oxide. Coatings of different thicknesses were analyzed. The researchers [25] found that the thickness of the oxide layer influenced the thickness of the lubricating film, formed by the lubricant containing the ZDDP additive. The results discussed above were in agreement with the Puhan and Reddyhoffs thesis that the surface layer influenced the tribochemical reactions of the ZDDP additive.

## 5. Conclusions

The following are conclusions drawn from the experimental results:

1. The effect of the interaction between ZDDP and CNTs was clearly visible; CNTs contributed to higher linear wear when a-C:H coated steel discs were used and lower linear wear when uncoated ones were employed.
2. The DLC coating significantly changed the mechanism of the antiwear action of the lubricant.

In the case of uncoated steel, the presence of ZDDP resulted in lower wear (smaller wear scars), whereas the addition of CNTs had an opposite effect: the area of the wear scars was bigger.

For a-C:H coated steel, there was an increase in wear when the lubricant was mixed with the ZDDP additive. The presence of CNTs in the lubricant, on the other hand, led to a considerable reduction in wear.

3. Opposite results were observed when linear wear was measured.

In the case of a-C:H coated discs, the antiwear properties of the lubricant containing ZDDP were higher at the beginning of the tribological tests, but they declined considerably after some time; the addition of CNTs eliminated the effect of wear prevention, and the wear was the highest at the end.

When the uncoated discs were tested in the presence of ZDDP, there was a decrease in wear; the addition of CNTs enhanced the antiwear effect of ZDDP.

4. The EDS analysis showed that the higher the concentration of Zn on the ball surface, the lower the wear; this relationship was less obvious for the a-C:H coated steel discs than for the uncoated ones.
5. The wear scar area was not dependent on the concentrations of the ZDDP triboreaction products but on the relationship between such concentrations on the ball surface and those observed on the disc surface. Much higher concentrations of the ZDDP triboreaction products on the ball surface led to a decrease in the wear scar area. This was most likely due to energy transfer.

The findings suggested that the mechanism of action of carbon nanotubes under friction conditions was more tribochemical in nature than mechanical (rolling, sliding), as postulated in [26]. The experimental results presented in this paper confirmed the thesis illustrated in Figure 1, which states that:

- CNTs enhance the transport of energy to the molecules of the lubricating additive (ZDDP);
- this energy influences the ZDDP tribochemical reactions;
- the structure and properties of the surface layer of a solid element in a tribological system play an important role in energy transfer.

The results of this study are preliminary. The research will be continued to collect more experimental data necessary to formulate a relationship between the surface layer structure and the rate of the tribochemical reactions of the antiwear additives, such as ZDDP.

## Figures and Tables

**Table 1:** Properties of PAO8.

Properties	Unit of Measurement	Value	Method
Specific gravity at 15.6 °C	kg/m^3^	833	ASTM D4052
Kinematic viscosity at 100 °C 40 °C −40 °C	mm^2^/s	7.8 46.4 19,570	ASTM D445
Viscosity index		138	ASTM D2270

**Table 2:** Properties of the commercial zinc dithiophosphate (ZDDP) lubricant additive.

Properties	Unit of Measurement	Value	Method
Density (25 °C)	kg/m^3^	1160	ASTM D1298
Kinematic viscosity at 40 °C	mm^2^/s	150	ASTM D445
Zn content	% weight	9	-
P content	% weight	8.5	-
S content	% weight	16.5	-

**Table 3:** Materials tested.

Lubricant Composition	Lubricant Symbol
PAO8	PAO8
PAO8 + 1.5%(m/m) ZDDP	PAO8 + ZDDP
PAO8 + 0.005% (m/m) CNT	PAO8 + CNT
PAO8 + 1.5% (m/m) ZDDP + 0.005% (m/m) CNT	PAO8 + ZDDP + CNT

**Table 4:** Composition of HS6-5-2C steel.

Element	%
C	0.82–0.92
Mn	≥0.4
Si	≥0.5
P	≥0.03
S	≥0.03
Cr	3.5–4.5
Ni	≥0.4
Mo	4.5–5.5
W	6–7
V	1.7–2.1
Co	≥0.5
Cu	≥0.3

**Table 5:** Surface texture parameters for the uncoated steel discs and steel balls before and after the tribological tests.

(a) Uncoated Discs										
**Surface Texture Parameters**	**Before Test; Uncoated Disc**		**PAO8**		**PAO8 + ZDDP**		**PAO8 + CNT**		**PAO8 + ZDDP + CNT**	
	**Disc**	**Ball**	**Disc**	**Ball**	**Disc**	**Ball**	**Disc**	**Ball**	**Disc**	**Ball**
Sa (µm)	0.34	0.15	0.44	0.60	0.39	2.09	0.45	0.58	0.36	1.98
Sq (µm)	0.44	0.22	0.56	1.09	0.49	2.46	0.56	0.91	0.46	3.15
Sp (µm)	1.66	2.26	1.90	16.52	1.78	4.89	1.66	1.76	1.40	22.85
Sv (µm)	2.39	2.38	5.03	4.95	2.02	7.71	1.71	15.06	2.07	8.92
Sz (µm)	4.04	4.64	6.93	21.47	3.80	12.61	3.37	16.83	3.47	31.77
Ssk (µm)	−0.56	0.06	−0.32	1.20	−0.24	−0.14	−0.59	−3.39	−0.44	1.34
Sku (µm)	4.20	9.23	3.83	27.32	2.85	2.05	3.22	24.50	3.14	12.09
(b) a-C:H Coated Discs										
**Surface Texture Parameters**	**Before Test;** **a-C:H Coated Disc**		**PAO8**		**PAO8 + ZDDP**		**PAO8 + CNT**		**PAO8 + ZDDP + CNT**	
	Disc	Ball	Disc	Ball	Disc	Ball	Disc	Ball	Disc	Ball
Sa (µm)	0.59	0.15	0.64	5.64	0.56	6.24	0.58	5.26	0.57	3.31
Sq (µm)	0.80	0.22	0.82	6.77	0.74	7.47	0.71	6.43	0.75	4.66
Sp (µm)	4.49	2.26	2.87	17.55	6.47	16.78	2.61	12.79	2.28	8.08
Sv (µm)	4.16	2.38	3.70	16.41	4.09	20.71	1.49	19.29	4.79	19.04
Sz (µm)	8.65	4.64	6.56	33.96	10.57	37.49	4.10	32.09	7.07	27.12
Ssk (µm)	−1.19	0.06	−0.70	−0.28	−0.91	−0.11	−0.21	−0.58	−0.89	−1.42
Sku (µm)	6.14	9.23	3.54	2.23	5.07	2.33	2.53	2.59	4.50	5.54
